# Supplementary material for: Participatory practices at work change attitudes and behavior toward societal authority and justice
Source: Nat Commun. 2020 May 26;11:2633. doi: 10.1038/s41467-020-16383-6 (PMC7250830; doi:10.1038/s41467-020-16383-6)
Supplement: Supplementary file 1 — Supplementary Information [file 41467_2020_16383_MOESM1_ESM.pdf]

Supplementary Information

Participatory practices at work change attitudes and behavior

toward societal authority and justice

Sherry Jueyu Wu

Elizabeth Levy Paluck

Table of Contents

***Supplementary Note 1 – Participatory Meetings Training Protocol* 2**

***Supplementary Note 2 – Qualitative Observations* 6**

***Supplementary Note 3 – Randomization*.. 14**

***Supplementary Note 4 – Robustness checks with group level t-tests* 19**

***Supplementary Note 5 – Robustness checks with 62 groups (intact pairs).* 21**

***Supplementary Note 6 – Robustness checks including new workers.* 24**

***Supplementary Note 7 – Robustness checks for missing value imputation*.............................. 27**

***Supplementary Note 8 – Survey Materials*... 30**

Supplementary Note 1 – Participatory Meetings Training Protocol

Participatory meetings are comprised of three parts: discussion, group leader summary, and goal setting. Each part should be connected fluently and be treated as an organic entity.

Discussion leaders should start to gather people with the help of the group leaders starting 7:30am. Each meeting lasts around 20 minutes and should end before 8am. A discussion leader's duty is to facilitate the discussion, encourage workers to speak up and actively engage in solving production related issues jointly as a group. Discussion leaders should let workers talk for most of the time during the meeting, rather than the group leader or the discussion leader herself. While workers engage in active discussion and goal setting, discussion leaders will facilitate. For the best discussion facilitation, always use simple and everyday Mandarin that fits the workers' communication habits. Do not use any formal or written language expressions. In your first meetings, get a general impression of the group's dynamics such as the gender and age composition, whether people were talkative, and what people cared about the most. Use these insights to prepare for your next meetings' facilitation.

Discussion (around 15 minutes). A pre-arranged shuttle picks up and takes everyone to the factory before 7:30am. Each research assistant has to be on the production floor where her assigned group is located. As a discussion leader (or an observer in the control condition), we can never show up late. Greet your workers politely when you arrive on the production floor. As 7:30 approaches, start to gather workers in the group with its group leader. When every worker in the group arrives, ask them to gather in a circle and greet them warmly.

During the first meetings, discussion leaders initiate a round of self-introduction, such as names and how long the workers have been working in the factory. Discussion leaders set clear

expectations during the first meeting, and briefly repeat the expectations at the start of each subsequent meeting. The protocol for discussion leaders goes:

"My name is *Zhang Xiaohong*, and you can call me Xiaohong. I'm a student from *Soochow University*, and I am helping a professor with a project on work experience. From this week on, I will come every *Monday* to lead a discussion with you on work related issues during your regular morning meeting time, for a period of six weeks. We will discuss the problems you have experienced in work, and the aim is for you to work better! Our meetings are easy-going. We encourage everyone to speak up! Just voice out whatever's on your mind about your work, such as issues yesterday or in the past week, the difficulties you have at work, or things you think will help you and others. I may ask some questions, and there's no right or wrong answers. Whatever you share will be helpful for the group and for us. I will take some notes during the discussion for research purposes, but I will not show my notes or talk with anyone who's not in our project team, including the factory people."

In subsequent meetings, discussion leaders repeat the expectation, "as we all know, it's a meeting for us to share our opinions on production related issues. I'm here to discuss with you on how to work better, rather than testing you. No worry about being right or wrong. Just say whatever you think of about work and participate!"

As a warm-up for problem solving, discussion leaders can start with easy questions such as "what type of order are you all working on today?" and "what steps are each of you in charge of?". Discussion leaders prepare and facilitate two questions for the workers to discuss. The number of discussion questions is secondary to the depth of the discussion. Though the content of discussion is flexible as long as it is work-related, we do have a module of focus for each

week during the six-week intervention period. The module and suggested discussion questions are as follows:

Week 1: General feedback meeting, getting to know each other

Week 2: Production speed and quantity (e.g., how to work faster? What gestures and strategies are most efficient?)

Week 3: Quality control (e.g., how to avoid defects? If a defect occurs, how to most efficiently coordinate for repairing? How to self-examine that finished pieces are good before going through quality control?)

Week 4: Order switch (e.g. how to shorten the adaptation period when production orders change? How to deal with issues in this fast transition period?)

Week 5: Group coordination (e.g. how to increase group efficiency, such as the arrangement and transition of finished pieces? How to coordinate with the person before and after you?)

Work 6: Discussion topic tailored to specific groups (e.g. if a group's major concern is its production speed, then focus more on this topic, etc.)

Supervisor summary (0-3 minutes). As the pre-existing 20-minute morning meetings were led by the supervisors for managerial purposes, we left this time for them in case there are other important managerial issues group members need to know that cannot be conveyed during group discussion. The first author and the discussion leaders had reminded the supervisors to keep their post-discussion summary brief.

Goal setting (2-3 minutes). Towards the meeting's end, workers are encouraged to make individual goals on their daily production. Since orders are placed by customers and have tentative amounts and deadlines for production, the discussion leaders (research assistants) tell

the workers about the orders placed by customers and specifically how many pieces each order requires and how long they have before the suggested deadline. For example, if an order placed asks for 10,000 pieces within 20 days, then a worker or a group is expected to produce around 500 pieces daily if they spread production evenly across days. The calculation is simple enough to do for workers with a Chinese elementary school education. Instead of being assigned a fixed production goal daily, workers will be given all the relevant information and encouraged to come up with a daily production goal themselves. Each worker is given a small piece of paper to do simple calculation and asked to voice out their goals in front of their group members.

In the end, discussion leaders wrap up the meeting and remind them about the following week's participatory meeting.

Supplementary Note 2 – Qualitative Observations

About the factory.

The study took place in the Chinese branch of a multinational apparel manufacturer, which is the largest in employee size among all branches and is located in the eastern coastal area of China. Our study population, the factory workers, were mostly young women in their twenties or thirties[1] who migrated from rural China to the city. The factory is built on the edge of the city and far from the city center, in a location that is relatively inconvenient to reach by public transportation. Around half of the workers live in adjacent factory dorms and another half commute to work on a daily basis. From field interviews prior to the experiment, we find that these workers are eager to work, but have little education (most of them have not finished high school) or training that would allow them to get a high-skilled job in the city. Hence, they enter manual work in apparel manufacturing, which is regarded as labor-intensive and low in skill requirement. Compared with its competitors, the factory pays very well and its workers, although extremely busy, mostly receive a salary in the rank of the lower-middle class[2] in the city.

Qualitative findings from the pilot intervention,

We recount the lessons learned from the pilot intervention (labeled *a* to *d*), followed by a series of field-note episodes within these experimental meetings, which briefly sketched parts of the meeting flow and group dynamics. Rather than a unified narrative, the following entries are a series of unfolding actions taken from different meetings across time. We understand that

[1] The fact that the workers are mostly young village women with little education is very similar to the personnel composition in the Harwood factory in Lewin' time. Whereas Harwood had around 300 workers, the current factory has thousands of workers, with quite different manufacturing scale.

[2] The monthly wage of a typical worker in the experimental factory ranges from 3,000 Yuan ($483.82) to more than 7,000 Yuan ($1128.92). Some reference statistics: In 2013, the minimum wage per month in Beijing was 1,400 Yuan, about 24 percent of the 5,793 Yuan average monthly wage, as calculated by the municipal bureau of statistics. In Shanghai, the 2013 minimum wage was 1,620 Yuan, or 32.2 percent of the 5,036 Yuan average wage. Those are two of the highest income cities in China. The living standard of Suzhou is very close to Shanghai.

observer biases are hard to avoid in qualitative data collection (Emerson, Fretz, & Shaw, 1995), but tried to be as impartial as possible about our observations and writings.

(a) Workers were very quiet in the first treatment meetings, and gradually talked more and more as they got used to this participatory style of meetings.

In the first meeting with the quality control group, the workers automatically formed two strictly straight lines close to each other, one in front and one back, like soldiers in an army. The researcher told them to feel more relaxed and form a circle so that everyone could face each other. In response to this, nobody moved. The same thing happened in the first meeting with the embroidery group, where workers automatically lined up, with a sizeable distance (at least 6 feet) away from the researcher. The supervisor later told us that the workers were never required to stand in lines during meetings, but they had formed such a habit for some unknown reasons. We speculate that workers have internalized the authoritarian work style of the factory, including the hierarchical arrangements, and were ready to follow any rule of an authority without question.

Workers were reluctant to speak up in the first meetings, and refrained even from saying their names, as demonstrated in the following excerpts from field notes:

> [1st meeting with the embroidery group, July 1]
>
> *I*[3] *started the meeting with self-introduction and went around the circle asking for their names. A silence. They were reluctant to talk. The girl standing in the middle of the workers facing me said "let's start with supervisor Wang." Wang said I had already met her. A short silence and I asked the person standing right next to me to start the self-*

[3] Taken from field notes. "I" refers to the first author.

introductions. The girl looked down to the ground and quietly said her name. Then one after another. I repeated each of their names, making sure I got them right.

[1st meeting with the packing group, July 7]

"What are you working on today as a group?" I asked.

The workers looked at each other and nobody spoke. I encouraged them to speak up. A woman raised her hand and said they were packing clothes. The others murmured.

However, workers adapted to the participatory style at a faster rate than we had expected. For example, at the second meeting with the embroidery group, the workers started to smile and greet the researcher when she came in, and actively discussed the June salary payment[4]. During the third and fourth meetings, most group members voluntarily expressed their opinions on production-related issues such as difficulty encountered with a new order, and how to develop good gestures with a machine.

(b) A participatory meetings intervention was the most effective when the tasks involved some level of collaboration between workers.

One question that remained unclear in Lewin's work was whether a participatory meetings intervention was equally applicable to every work group. As a large part of the intervention treatment focuses on eliciting information exchange among workers on production related issues, we suspected the intervention would be most relevant for the groups which require collaborative tasks between group members, or for which an individual worker's performance is affected by and will affect the performance of her coworkers. It was indeed what we found in our pilot intervention. In groups that require collaboration between workers, like the sewing, packing,

[4] The salary payment day is on the 7th of each month.

and cutting groups, workers expressed many constructive suggestions on what they need from the workers next to them to help themselves work faster and better, and what they could do to help their coworkers work faster and better. They had never thought of or had the chance to openly discuss working strategies like those. However, the discussion about group collaboration was not very successful for the embroidery and quality control groups, whose tasks did not necessitate collaboration between workers, as illustrated in the following episodes:

[1st meeting with the embroidery group, July 1]

"Are you working on short-sleeve or long-sleeve clothes? What kind of things are you all working on as a group today?" I asked.

"Each of us works on different things." A worker said.

"What are each of you working on today?" I asked a second question.

"We all work on different things. Different things every day. We follow the supervisor's assignments."

"What are some of the strategies you've used for this task? Do you mind sharing with others? You know as a group, we need collaboration."

A silence.

"Collaboration is not needed. We work on different things." A woman quietly responded.

(c) The discussion flow was much more fluent for the groups whose workers' jobs were interdependent. Any work problem could be easily turned into a discussion after the first meeting.

The sewing division is the factory's largest and most labor-intensive. Workers are organized into 20-30 person groups which work on a specific order placed by companies all over the world. For example, a group may specifically work on a purple baby one-piece while another group works specifically on a blue dress during a certain time period. Each worker in a group is assigned a step in the apparel production processes and tends to repeat the same step until the whole order is completed. As a group is vertically integrated, an individual's work performance might affect the workers after her, even though their salary earnings only depend on individual piece rates. Nevertheless, the optimal strategy is for the group to achieve its maximum productivity so that everyone can have a stable high level of output, rather than for each individual to maximize her own profit (which results in a fluctuation of individual productivity because an individual cannot produce faster when prior steps are not finished). Coordination issues become more prominent when there is a production order switch. One problem came with new cloth patches, and inefficient coordination between workers and between different divisions. Workers complained about frequent order switches because they thought their salaries would suffer. Workers expressed grudges in the discussions. As one put it: "I can't work fast with new tasks. And I can't work fast unless the person before me works fast." Workers looked surprised when they heard that actually everyone shared the same problem. A solution might be as simple as help to unpack patches:

> [2nd meeting with Friday's sewing group, July 10]
>
> *A girl standing in the middle, who is in charge of the first step of the work process, said: "It would be great if the person after me or someone else will lend me a hand to carry the materials from carts to my working desk. The materials are too heavy for me and it slows me and also the group down."*

The woman after her nodded immediately and said she never noticed that the material-moving was slowing their performance. Another worker said she hoped others would help her to do a few pieces when she could not finish all of them in time.[5] For the sewing groups, any production-related issue could be developed into a discussion. Another example follows:

[2nd meeting with Thursday's sewing group, July 9]

"We just changed new machines four days ago. It's hard to adapt to them."

"I'm a lot slower under the new machine and I don't like it."

"The technicians are not very responsive to our needs. I asked him several times to adjust my machine, but he's slow."

"I have that problem too!"

"Me too."

These responses were prompted by the question, "what are some of your production problems that you've encountered this week?". It turned out that the sewing departments just changed new machines the previous week. The new machines were supposed to be better and safer than the old ones. But workers did not like them. Even though the factory had organized a lecture series from a technician on using the new machines, workers still had many problems unsolved. In the meeting workers discussed the problems they had encountered with the new machines. Hearing their voice, the supervisor focused on these problems in her supervisor

[5] Every piece that is done by a certain individual will be counted as her own production, no matter if the pieces are assigned to her or to others. Thus helping is not "free." However, from observation and interviews, workers seldom help others unless the group leader intervenes either because they did not know others need a help or they thought helping others waste their time.

summary part of the meeting, such as how to avoid leaking machine oil and how to communicate problems to the technicians.

(d) Putting questions in context and activating social roles elicited more responses than asking about individual experiences alone. Asking a question in an utmost concrete way was the most effective in getting responses from workers.

At the first several meetings, we encountered a problem that the workers found it difficult to describe "working strategies" in detail. Some workers thought on a very abstract level and said there were no specific strategies and everything came with some working experience. Several women mentioned that people would know the right gestures with experience but were unable to describe the process further. The researcher asked what a good gesture looked like and how to develop it. Again, the workers were unable to describe it in detail. However, when we put the question in the current context of the group and asked the question in another way, workers were able to understand the question in a concrete level and start the discussion:

[4rd meeting with Saturday's sewing group, July 25]

The group switched order from producing a summer dress to a winter baby outfit at the beginning of this week. In the discussion, everyone said something about why switching tasks was hard for them. "We cannot work fast with new tasks. It takes time." Several workers said. I asked whether everyone slowed down during order switch and they all said yes, but some workers took less time to get used to the new tasks because they have "good gesture."

"What is a good gesture? Do you mind sharing with the rest of us?"

A silence. Some people appeared to be thinking.

"A gesture is something that comes naturally." A worker said.

"Previously you said more experienced workers tend to develop good gestures. For those of you who have been here long enough, how would you teach the newer workers here? What would you do if a new worker asks you about the good gestures?"

A woman then walked close to the machine and showed how she used a mold to help sew a squared patch onto the front of an outfit seamlessly.

Apparently, the second way of asking the same question on "good gestures" was more effective as it created a concrete scenario for the workers to act upon, in particular when the social role of a "more experienced worker" in relation to a "new worker" was activated. Similarly, when we discussed why product defects occurred and how to avoid them, the workers' first reactions were: "*I should be more careful," "It comes with working experience. With more working experience, workers know how to avoid the defects,*" as two workers said during the discussions. But when the researcher pointed to a specific defective piece and asked how it occurred and how to repair it, workers focused the discussions on working strategies to fix that specific piece as well as other more general issues on product defects.

Workers were not used to thinking analytically about problems unless provided a concrete example or scenario. It may reflect a cross-cultural difference in people's thinking style (Nisbett, Peng, Choi, & Norenzayan, 2011). The factory workers are used to thinking holistically rather than analytically. Thus in the actual intervention, we always asked a question in the most concrete way possible and activated the context and social relations surrounding the target question to help workers engage in extended discussions.

Supplementary Note 3 – Randomization

We conducted Study 1 at a multinational textile factory in China. We sampled all work groups (N = 65) from the factory's sewing departments where workers are organized in groups. Employees in the sewing work groups work on their own tasks, which are related to their coworkers' tasks. For example, one worker may be in charge of sewing the sleeves of a hoodie while another is in charge of sewing the hood pieces. Each work group has its own supervisor who oversees group members' work. The factory requires all work groups to have a 20-minute morning meeting before the start of each workday, in which the supervisor summarizes the previous day's work performance, recommends individual and group work strategies, and announces goals for individual workers. Workers rarely transfer to a different group after they are hired. Individuals in all groups provided informed consent during a recruitment phase one month before the experiment's commencement (no refusals were observed).[6]

We randomly assigned the 65 work groups ($N_{workers}$ = 1,752; 93.6% female; mean age = 32.5 years, ranging from 18 to 53) to participate in a weekly morning participatory meeting (referred to as *participatory meetings condition* or *treatment condition*), or to have an observer attend the usual morning meeting (referred to as *observer condition* or *control condition*) once per week for six weeks.[7] To randomize, we used a non-bipartite matching scheme (Beck, Lu, & Greevy, 2015) to balance and minimize observable differences between the groups ex ante. Within each department, work groups were matched based on their group size, average worker

[6] We made oral public announcements in the sewing departments to invite all sewing workers to a study called "worker experience in the factory," with the cooperation of the factory's human resource department. Workers were specifically told that "researchers are not part of the factory but are coming to learn management practices and offer new technologies on work-related issues. All of you are invited to take part[…]Participation is completely voluntary."

[7] The building structure allows for little communication between sewing departments and among work groups. Workers spend most of their time in their own group's working area on the production floor during work, and have little communication with other groups during and after work. Thus, we have few concerns about spillover of treatment to control groups.

productivity, normal working hours, and overtime working hours. We took into account qualitative comments from departmental supervisors on the leadership style of each supervisor to fine-tune the group matches prior to randomization (see matching code below). When groups were paired within departments, we randomly assigned one group in each pair to the *participatory meetings condition*, and the other to the *observer condition*. We assigned the three groups that did not achieve a match within their departments to the observer condition, as desired by the factory (results are robust to the exclusion of these three unmatched groups; see the supplementary materials). The final sample consists of 31 treatment groups ($N_{workers}$ = 868) and 34 control groups ($N_{workers}$ = 881).

In Study 2, forty academic departments' administrative staff groups (out of 70 total) at a university in the United States agreed to participate in the study during the recruitment phase. Each group was comprised of an academic manager (the supervisor) and staff members who directly report to the manager (the workers). Supervisors agreed to participate in the study, and we moved forward with their participation if a majority of their staff also consented to participate. The median size of the administrative group was 6. The staff members' work is relatively independent, including job roles such as graduate and undergraduate administrators, finance managers, and event coordinators.

As in Study 1, we randomly assigned the 40 administrative groups to participate in a weekly morning participatory meeting (*participatory meetings* or *treatment condition)* or continue with their status-quo meetings (*control condition*). Supervisors and workers provided written informed consent before the experiment's commencement. To randomize, we used the same non-bipartite matching scheme in Study 1. Staff groups were matched based on their academic division (Natural Sciences, Social Sciences, Humanities, or Engineering), program

category (full-degree programs or interdisciplinary centers), staff headcounts, faculty headcounts, and initial level of enthusiasm shown by their RSVP and attendance at our recruitment events. When departments were paired, we randomly assigned one department in each pair to the participatory meetings condition, and the other to the control condition. Eight staff groups ($N_{treatment}$ = 5, $N_{control}$ = 3) dropped out of the study after randomization but before any data collection.[8] The final sample consists 32 administrative groups or 172 individual staff members (78% female, 22% male; 80% identified as White or European-American; mean age = 50 years, ranged from 25 to 88).

[8] Reasons for attrition were departmental staff changes or not having enough time in the current academic semester.

Supplement Table 1. Balance check in Study 1. The balance test showed there was no significant differences between participatory meetings and observer condition for any pre-treatment characteristics. Omnibus p = 0.40.

Note: $^{*}p < 0.05$; $^{**}p < 0.01$; $^{***}p < 0.001$.

	Condition Assignment
Education	0.303
	(0.219)
DeptA3	-0.647
	(1.164)
DeptB3	-0.532
	(1.218)
DeptC2	-0.501
	(1.193)
DeptC3	-0.326
	(1.180)
DeptD2	-0.048
	(1.181)
DeptD3	-0.614
	(1.243)
Work experience	-0.095
	(0.052)
Baseline productivity	-0.0002
(first 6-week period)	(0.0002)
Baseline productivity	0.0002
(second 6-week period)	(0.0002)
Gender	0.363
	(0.304)
Age	-0.024
	(0.015)
Constant	0.852
	(1.344)

Non-bipartite matching codes (adapted from Jas Sekhon).

```
library("nbpMatching")
# Generate example data
my_data <- data.frame(x1 = rnorm(100),
                      x2 = rnorm(100),
                      x3 = rnorm(100),
                      x4 = rnorm(100))
# Extract variables we want to match on
match_data <- my_data[, c("x1", "x3", "x4")]
# Make distance matrix (can use `gendistance` function as well)
dist_mat <- as.matrix(dist(match_data))
# Construct matches
matches <- nonbimatch(distancematrix(dist_mat))
# Each row in `extracted_matches` is a paired-match
extracted_matches <- cbind(matches$halves$Group1.Row,
                           matches$halves$Group2.Row)
```

Supplementary Note 4 – Robustness checks with group level t-tests

Here, we replace all the causal inferences in the results section from multiple regressions with between-group t-tests, treating group as units of analysis.

Attitudinal Changes toward Authority and Justice

General attitudes toward authority. The mean score of attitudes toward authority for the whole sample was 4.05 (SD = 0.37). This value accords with the "slightly agree" point of the scale, which indicates that, on average, workers tended to slightly agree with statements asserting complete obedience and respect for authority without question. However, workers in the participatory meetings condition reported significantly lower score in attitudes toward authority (M = 3.87, SD = 0.32) than workers in the observer condition (M = 4.23, SD = 0.33, t = 4.36, p < .001, d = 1.08). Participatory meetings changed participants' attitudes toward general authority such that treatment workers registered as less authoritarian on a traditional scale of authoritarianism.

These results were consistent when we analyzed each item within the index. One month after the end of the experiment, treatment workers who took part in these brief participatory meetings were significantly less likely to endorse obedience and respect for authority as the most important virtues children should learn (t = 2.86, p < .006, d = 0.71), less likely to agree everybody would be better off if people would talk less and work more (t = 5.04, p <.001, d = 1.26), and less likely to believe in supernatural power (t = 2.30, p = .020, d = 0.59).

Belief in a just world. For attitudes and perceptions in generalized justice, the mean score for the whole sample was 3.98 (SD = 0.23). This value is also just below the "slightly agree" point of the scale, which indicates that, on average, workers tend to slightly agree with statements asserting belief in a just world. Workers in the participatory meetings condition

reported significantly lower score in belief on justice and fairness (M = 3.86, SD = 0.22) than workers in the observer condition who on average slightly agree on a just world belief (M = 4.10, SD = 0.16, $t = 4.93$, $p < .001$, $d = 1.24$). The results were also consistent for each individual item in the scale. There was no difference among workers with different demographics.

Perceived intergroup conflict. Participants in the treatment and control group did not differ in their perceived conflict relations between the rich people and normal people (M_{PM} = 3.56, SD = 0.22; M_O = 3.50, SD = 0.23; $p = .10$, n.s.), or between the capitalists and the working class (M_{PM} = 3.55, SD = 0.27; M_O = 3.29, SD = 0.17; $p = .26$, n.s.). However, workers in the participatory meetings condition reported more conflict between managers and workers in Chinese society than workers in the observer condition (M_{PM} = 3.55, SD = 0.27; M_O = 3.29, SD = 0.17; $t = 4.70$, $p < .001$, $d = 1.19$).

Participation outside of work. Workers in the participatory meetings condition reported higher levels of participation outside of work (M = 4.39, SD = 0.19) than workers in the observer condition (M = 4.21, SD = 0.19), $t = 3.78$ $p < .001$, $d = 0.94$. The same pattern was observed for the two spheres of participation outside of work – engagement with politics and with family life. Workers in the participatory meetings condition reported significantly higher interest in participation in politics (M = 4.06, SD = 0.32) than workers in the observer conditions (M = 3.80, SD = 0.34), $t = 3.33$, $p = .001$, $d = 0.83$. Likewise, workers in the participatory meetings condition reported significantly higher participation in family and social life (M = 4.54, SD = 0.19) than workers in the observer condition (M = 4.41, SD = 0.21), $t = 2.54$, $p = .014$, $d = 0.63$. Robustness check without the three unmatched groups in the observer contidion.

Supplementary Note 5 – Robustness checks with 62 groups (intact pairs).

General attitudes toward authority. The mean score of attitudes toward authority (□ = .52) for the whole sample was 4.03 (*SD* = 0.36). This value accords with the "slightly agree" point of the scale, which indicates that, on average, workers tended to slightly agree with statements asserting complete obedience and respect for authority without question. However, workers in the participatory meetings condition reported significantly lower scores in attitudes toward authority (*M* = 3.86, *SD* = 0.32) than workers in the observer condition (*M* = 4.19, *SD* = 0.32, β = -0.35, *CI* = [-0.51, -0.20], *SE* = 0.08, $p < .001$). Participatory meetings changed participants' attitudes toward general authority such that treatment workers registered as less authoritarian on a traditional scale of authoritarianism.

These results were consistent when we analyzed each item within the index. One month after the end of the experiment, treatment workers who took part in these brief participatory meetings were significantly less likely to endorse obedience and respect for authority as the most important virtues children should learn (M_{PM} = 4.47, SD = 0.41, M_C = 4.77, SD = 0.46; β = -0.35, CI = [-0.56, -0.15], SE = .11, $p < .001$), less likely to agree everybody would be better off if people would talk less and work more (M_{PM} = 4.40, SD = 0.40, M_C = 4.84, SD = 0.36; CI = [-0.66, -0.31], SE = .09, β = -0.49, $p < .001$), and less likely to have complete faith in supernatural power (M_{PM} = 2.71, SD = 0.44, M_C = 2.95, SD = 0.44; CI = [-0.44, -0.02], SE = .11, β = -0.23, $p = .036$).

Belief in a just world. For attitudes and perceptions in generalized justice (□□□□□□□□□, the mean score for the whole sample was 3.98 (*SD* = 0.23). This value is slightly below the "slightly agree" point of the scale, which indicates that, on average, workers tended to slightly agree with statements asserting belief in a just world. Workers in the

participatory meetings condition reported significantly lower score in belief in a just world (*M* = 3.86, *SD* = 0.22) than workers in the observer condition who on average slightly agree with a just world belief (*M* = 4.11, *SD* = 0.16; β = -0.27, *CI* = [-0.35, -0.18], *SE* = 0.04, $p < .001$). The results were also consistent for each individual item in the scale. There was no difference among workers with different demographics.

Perceived intergroup conflict. Participants in the treatment and control group did not differ in their perceived conflict relations between the rich people and normal people (M_{PM} = 3.60, *SD* = 0.23; M_O = 3.50, *SD* = 0.27; *p* = .08, n.s.), or between the capitalists and the working class (M_{PM} = 3.56, *SD* = 0.22; M_O = 3.50, *SD* = 0.24; *p* = .22, n.s.). However, workers in the participatory meetings condition reported more conflict between managers and workers in Chinese society than workers in the observer condition (M_{PM} = 3.55, *SD* = 0.27; M_O = 3.29, *SD* = 0.17; β = 0.32, *CI* = [0.21, 0.42], *SE* = 0.05, $p < .001$). Overall, treatment workers reported a higher level of intergroup conflict than control workers on average (□ = .62; M_{PM} = 3.57, *SD* = 0.20; M_O = 3.43, *SD* = 0.16; β = 0.17, *CI* = [.08, .26], *SE* = 0.05, $p < .001$).

Participation outside of work. Workers in the participatory meetings condition reported higher levels of participation behavior outside of work (□ = .37; *M* = 4.39, *SD* = 0.19) than workers in the observer condition (*M* = 4.21, *SD* = 0.20), β = 0.17, *SE* = 0.05, *CI* = [0.08, 0.27], $p < .001$. The same pattern was observed for the two spheres of off-work participation behavior–engagement with politics and with family and social life. Workers in the participatory meetings condition reported significantly higher interest in participation in politics (*M* = 4.07, *SD* = 0.32) than workers in the observer conditions (*M* = 3.82, *SD* = 0.34), β = 0.27, *CI* = [0.10, 0.43], *SE* = 0.08, *p* = .001). Likewise, workers in the participatory meetings condition reported significantly

more participation in family and social life (*M* = 4.54, *SD* = 0.19) than workers in the observer condition (*M* = 4.41, *SD* = 0.22), β = 0.12, *CI* = [.02, .22], *SE* = .05, *p* = .015.

Joint Significance Test

Because we tested the average treatment effects on multiple dependent variables, we conducted a joint significance test on the null hypothesis that the coefficients on average treatment effects from all the multiple regressions are jointly nonsignificant. As predicted, there was a jointly significant difference of the average treatment effects between workers in the participatory meetings condition and observer condition, $F(1, 58) = 7.59$, $p < .001$.

As a robustness check, the multivariate analysis of variance (MANOVA) was conducted to assess condition difference on all dependent variables recorded. The multivariate effect was significant by conditions, $F(1, 63) = 7.89$, $p < .001$. Thus, we conclude that participatory meetings significantly changed workers' attitudes compared with workers in the observer condition.

Supplementary Note 6 – Robustness checks including new workers.

Attitudinal Changes toward Authority and Justice

General attitudes toward authority. The mean score of attitudes toward authority (□ = .52) for the whole sample was 4.00 (*SD* = 0.37). This value accords with the "slightly agree" point of the scale, which indicates that, on average, workers tended to slightly agree with statements asserting complete obedience and respect for authority without question. However, workers in the participatory meetings condition reported significantly lower scores in attitudes toward authority (*M* = 3.84, *SD* = 0.33) than workers in the observer condition (*M* = 4.14, *SD* = 0.35, β = -0.39, *CI* = [-0.54, -0.24], *SE* = 0.08, p < .001). Participatory meetings changed participants' attitudes toward general authority such that treatment workers registered as less authoritarian on a traditional scale of authoritarianism.

These results were consistent when we analyzed each item within the index. One month after the end of the experiment, treatment workers who took part in these brief participatory meetings were significantly less likely to endorse obedience and respect for authority as the most important virtues children should learn (M_{PM} = 4.41, SD = 0.43, M_C = 4.64, SD = 0.47; β = -0.39, CI = [-0.59, -0.19], SE = .10, p < .001), less likely to agree everybody would be better off if people would talk less and work more (M_{PM} = 4.35, SD = 0.40, M_C = 4.71, SD = 0.44; CI = [-0.70, -0.35], SE = .09, β = -0.53, p <.001), and less likely to have complete faith in supernatural power (M_{PM} = 2.76, SD = 0.40, M_C = 3.06, SD = 0.43; CI = [-0.48, -0.06], SE = .11, β = -0.27, p = .013).

Belief in a just world. For attitudes and perceptions in generalized justice (□□□□□□□□, the mean score for the whole sample was 3.90 (*SD* = 0.24). This value is slightly below the "slightly agree" point of the scale, which indicates that, on average, workers

tended to slightly agree with statements asserting belief in a just world. Workers in the participatory meetings condition reported significantly lower score in belief in a just world ($M = 3.83$, $SD = 0.24$) than workers in the observer condition who on average slightly agree with a just world belief ($M = 3.97$, $SD = 0.22$; $\beta = -0.25$, $CI = [-0.33, -0.17]$, $SE = 0.04$, $p < .001$). The results were also consistent for each individual item in the scale. There was no difference among workers with different demographics.

Perceived intergroup conflict. Participants in the treatment and control group did not differ in their perceived conflict relations between the rich people and normal people ($M_{PM} = 3.60$, $SD = 0.22$; $M_O = 3.51$, $SD = 0.24$; $p = .10$, n.s.), or between the capitalists and the working class ($M_{PM} = 3.56$, $SD = 0.22$; $M_O = 3.51$, $SD = 0.21$; $p = .25$, n.s.). However, workers in the participatory meetings condition reported more conflict between managers and workers in Chinese society than workers in the observer condition ($M_{PM} = 3.55$, $SD = 0.26$; $M_O = 3.29$, $SD = 0.18$; $\beta = 0.31$, $CI = [0.21, 0.42]$, $SE = 0.05$, $p < .001$). Overall, treatment workers reported a higher level of intergroup conflict than control workers on average (□ = .62; $M_{PM} = 3.57$, $SD = 0.20$; $M_O = 3.44$, $SD = 0.15$; $\beta = 0.16$, $CI = [.07, .25]$, $SE = 0.04$, $p < .001$).

Participation outside of work. Workers in the participatory meetings condition reported higher levels of participation behavior outside of work (□□= .37; $M = 4.38$, $SD = 0.19$) than workers in the observer condition ($M = 4.21$, $SD = 0.18$), $\beta = 0.19$, $SE = 0.05$, $CI = [0.09, 0.28]$, $p < .001$. The same pattern was observed for the two spheres of off-work participation behavior–engagement with politics and with family and social life. Workers in the participatory meetings condition reported significantly higher interest in participation in politics ($M = 4.07$, $SD = 0.32$) than workers in the observer conditions ($M = 3.80$, $SD = 0.33$), $\beta = 0.30$, $CI = [0.15, 0.46]$, $SE = 0.08$, $p < .001$). Likewise, workers in the participatory meetings condition reported significantly

more participation in family and social life (*M* = 4.54, *SD* = 0.20) than workers in the observer condition (*M* = 4.41, *SD* = 0.20), β = 0.12, *CI* = [.03, .21], *SE* = .05, *p* = .007.

Joint Significance Test

Because we tested the average treatment effects on multiple dependent variables, we conducted a joint significance test on the null hypothesis that the coefficients on average treatment effects from all the multiple regressions are jointly nonsignificant. As predicted, there was a jointly significant difference of the average treatment effects between workers in the participatory meetings condition and observer condition, $F(1, 58) = 5.30$, $p < .001$.

As a robustness check, the multivariate analysis of variance (MANOVA) was conducted to assess condition difference on all dependent variables recorded. The multivariate effect was significant by conditions, $F(1, 63) = 5.63$, $p < .001$. Thus, we conclude that participatory meetings significantly changed workers' attitudes compared with workers in the observer condition.

Supplementary Note 7 – Robustness checks for missing value imputation.

Attitudinal Changes toward Authority and Justice

General attitudes toward authority. The mean score of attitudes toward authority (□ = .52) for the whole sample was 4.05 (*SD* = 0.35). This value accords with the "slightly agree" point of the scale, which indicates that, on average, workers tended to slightly agree with statements asserting complete obedience and respect for authority without question. However, workers in the participatory meetings condition reported significantly lower scores in attitudes toward authority (*M* = 3.87, *SD* = 0.32) than workers in the observer condition (*M* = 4.20, *SD* = 0.30, β = -0.35, *CI* = [-0.48, -0.21], *SE* = 0.07, $p < .001$). Participatory meetings changed participants' attitudes toward general authority such that treatment workers registered as less authoritarian on a traditional scale of authoritarianism.

These results were consistent when we analyzed each item within the index. One month after the end of the experiment, treatment workers who took part in these brief participatory meetings were significantly less likely to endorse obedience and respect for authority as the most important virtues children should learn (M_{PM} = 4.48, SD = 0.39, M_C = 4.77, SD = 0.41; β = -0.32, CI = [-0.49, -0.15], SE = .09, $p < .001$), less likely to agree everybody would be better off if people would talk less and work more (M_{PM} = 4.42, SD = 0.38, M_C = 4.85, SD = 0.34; CI = [-0.60, -0.28], SE = .09, β = -0.44, $p <.001$), and less likely to have complete faith in supernatural power (M_{PM} = 2.72, SD = 0.43, M_C = 2.98, SD = 0.44; CI = [-0.45, -0.10], SE = .09, β = -0.28, $p = .002$).

Belief in a just world. For attitudes and perceptions in generalized justice (□□□□□□□□, the mean score for the whole sample was 3.98 (*SD* = 0.21). This value is slightly below the "slightly agree" point of the scale, which indicates that, on average, workers

tended to slightly agree with statements asserting belief in a just world. Workers in the participatory meetings condition reported significantly lower score in belief in a just world (*M* = 3.87, *SD* = 0.21) than workers in the observer condition who on average slightly agree with a just world belief (*M* = 4.10, *SD* = 0.15; β = -0.22, *CI* = [-0.30, -0.15], *SE* = 0.04, $p < .001$). The results were also consistent for each individual item in the scale. There was no difference among workers with different demographics.

Perceived intergroup conflict. Participants in the treatment and control group did not differ in their perceived conflict relations between the rich people and normal people (M_{PM} = 3.60, *SD* = 0.22; M_O = 3.53, *SD* = 0.19; *p* = .16, n.s.), or between the capitalists and the working class (M_{PM} = 3.56, *SD* = 0.21; M_O = 3.49, *SD* = 0.21; *p* = .14, n.s.). However, workers in the participatory meetings condition reported more conflict between managers and workers in Chinese society than workers in the observer condition (M_{PM} = 3.55, *SD* = 0.25; M_O = 3.30, *SD* = 0.16; β = 0.28, *CI* = [0.18, 0.37], *SE* = 0.05, $p < .001$). Overall, treatment workers reported a higher level of intergroup conflict than control workers on average (□ = .62; M_{PM} = 3.57, *SD* = 0.19; M_O = 3.44, *SD* = 0.14; β = 0.14, *CI* = [.06, .22], *SE* = 0.04, $p < .001$).

Participation outside of work. Workers in the participatory meetings condition reported higher levels of participation behavior outside of work (□ = .37; *M* = 4.38, *SD* = 0.17) than workers in the observer condition (*M* = 4.23, *SD* = 0.16), β = 0.15, *SE* = 0.04, *CI* = [0.07, 0.23], $p < .001$. The same pattern was observed for the two spheres of off-work participation behavior–engagement with politics and with family and social life. Workers in the participatory meetings condition reported significantly higher interest in participation in politics (*M* = 4.06, *SD* = 0.31) than workers in the observer conditions (*M* = 3.82, *SD* = 0.30), β = 0.24, *CI* = [0.11, 0.38], *SE* = 0.07, $p < .001$). Likewise, workers in the participatory meetings condition reported significantly

more participation in family and social life (*M* = 4.54, *SD* = 0.17) than workers in the observer condition (*M* = 4.44, *SD* = 0.18), β = 0.11, *CI* = [.02, .19], *SE* = .04, *p* = .011.

Joint Significance Test

Because we tested the average treatment effects on multiple dependent variables, we conducted a joint significance test on the null hypothesis that the coefficients on average treatment effects from all the multiple regressions are jointly nonsignificant. As predicted, there was a jointly significant difference of the average treatment effects between workers in the participatory meetings condition and observer condition, $F(1, 58) = 8.06$, $p < .001$. As an additional robustness check, we conducted a multivariate analysis of variance (MANOVA) to assess treatment differences on all dependent variables recorded. The multivariate effect was significant by treatment, $F(1, 63) = 8.45$, $p < .001$, partial [illegible]. Thus, we conclude that participatory meetings significantly changed workers' attitudes compared with workers in the observer condition.

Supplementary Note 8 – Survey Materials

In Study 1, due to workers' time constraints, we conducted the survey during the 1-hour meal time and provided a free meal as incentive. On average, 4-5 groups (not segregated by treatment assignment) gathered for a mealtime survey session. The survey was described as "part of a research project that investigates worker experiences in the factory and in the larger society." Workers were assured that there were no right or wrong answers, that the survey was confidential, and specifically that researchers would not share individual answers with the factory management. To further ensure confidentiality, participants put their completed answer sheets in a sealed envelope and put their envelope in a transparent box with all other surveys. No identifiable survey data were shared with the factory, as agreed in advance of the study.

A considerable proportion of the factory worker population is illiterate, so a standard written survey was not be feasible. Prior in-depth cognitive interviews with an independent sample of factory workers guided the development of our survey instrument, which combined oral questioning in a group setting with workers checking boxes on individual answer sheets. Researchers read each survey question aloud, and participants marked their responses on answer sheets (featuring boxes for "yes" or "no", or numbers from 1-6 on a Likert scale of agreement that was well-explained in advance). This procedure obviated the need to read or write Mandarin characters. Participants were encouraged to interrupt the researchers for question clarification, but were not allowed to discuss their answers or look around each other's answer sheets during the session.

Supplementary Table 2. Study 1 survey items。

Category	Item
Participation outside of work (□ = .37)	How much impact do you feel government policies have on your daily life?
	How often do you follow news about politics, e.g. in the daily newspaper, on television, on the radio?
	How’s your relationship with your family?
	How often have you participated in your family’s decision making lately? (never; sometimes; often; always)
	How much influence do you intend to have on your kids or your future kids?
	How often do you socialize with your teammates off-work these days?
Attitudes toward authority (□ = .54)	Obedience and respect for authority are the most important virtues children should learn.
	If people would communicate less and work more, everybody would be better off.
	Every person should have complete faith in some supernatural power whose decisions he obeys without question.
Belief in a just world (□ = .32)	Although evil men may hold political power for a while, in the general course of history good wins out.
	It is often impossible for a person to receive a fair trial in China.
	By and large, people deserve what they get.

	Do you think most people would try to take advantage of you if they got a chance, or would they try to be fair? Please show your response on this card, where 1 means that “people would try to take advantage of you,” and 6 means that “people would try to be fair”.
Conflict	In your mind, to what extent do the following social groups have conflict with each other?<br><br>The rich and the "ordinary"
	The capitalists and the working class
	Managers and workers in big Chinese society

Supplementary Table 3. Study 2 survey items.

Category	Item
Attitudes toward authority (□ = .55)	Obedience and respect for authority are the most important virtues children should learn.
	If people would communicate less and work more, everybody would be better off.
	Every person should have complete faith in some supernatural power whose decisions he obeys without question.
Belief in a just world (□ = .21)	Although evil men may hold political power for a while, in the general course of history good wins out.
	It is often impossible for a person to receive a fair trial in the US. (R)
	By and large, people deserve what they get.

Supplementary Table 4. *Study 1 General Attitudes Toward Authority*.

	Dependent variable:			
	Average	Individual items		
		(1)	(2)	(3)
Participatory meetings	-0.390***	-0.381***	-0.524***	-0.267*
	(0.079)	(0.102)	(0.090)	(0.109)
Gender	0.188	0.235	0.152	0.143
	(0.115)	(0.122)	(0.168)	(0.198)
Marital status	-0.139	-0.101	-0.008	-0.303
	(0.258)	(0.210)	(0.287)	(0.478)
Number of children	0.037	-0.018	0.039	0.089
	(0.050)	(0.063)	(0.072)	(0.074)
Child at home	0.009	-0.073	0.037	0.092
	(0.085)	(0.079)	(0.090)	(0.158)
Origin	-0.163	-0.102	-0.181	-0.203
	(0.183)	(0.212)	(0.204)	(0.326)
Departmental fixed effects	YES	YES	YES	YES
Constant	4.402***	5.152***	4.989***	3.049***
	(0.248)	(0.304)	(0.241)	(0.381)

Note: *p<0.05; **p<0.01; ***p<0.001

Supplementary Table 5. *Study 1 Belief in a Just World.*

	Dependent variable:				
	Average	Individual items			
		(1)	(2)	(3)	(4)
condtreatment	-0.258***	-0.380***	-0.026+	-0.384***	-0.205*
	(0.041)	(0.064)	(0.076)	(0.092)	(0.083)
Male	0.114	0.026	0.250	0.247	-0.064
	(0.088)	(0.112)	(0.168)	(0.160)	(0.161)
Single	0.011	0.264	-0.130	-0.234	0.146
	(0.158)	(0.207)	(0.278)	(0.306)	(0.339)
Number of children	-0.024	0.022	0.017	-0.055	-0.064
	(0.034)	(0.060)	(0.073)	(0.056)	(0.070)
Child at home	-0.020	-0.053	0.081	-0.118	0.032
	(0.051)	(0.067)	(0.087)	(0.073)	(0.109)
Origin	-0.085	-0.036	0.113	-0.362	-0.052
	(0.114)	(0.178)	(0.173)	(0.192)	(0.227)
	YES	YES	YES	YES	YES
Constant	4.224***	5.121***	2.314***	5.074***	4.354***
	(0.138)	(0.232)	(0.233)	(0.247)	(0.268)
Note:					*p<0.05; **p<0.01; ***p<0.001

Supplementary Table 6. *Study 2 Attitudes Toward General Authority.*

	Dependent variable:			
	Average	Individual items		
		(1)	(2)	(3)
Participatory meetings	-0.439*	-0.731*	-0.335+	-0.315
	(0.209)	(0.316)	(0.269)	(0.247)
Male	0.027	0.001	0.251	-0.158
	(0.245)	(0.379)	(0.276)	(0.400)
Age	-0.004	-0.009	-0.002	-0.002
	(0.008)	(0.014)	(0.011)	(0.009)
Racial minority	0.696**	1.116*	0.271	0.707*
	(0.265)	(0.437)	(0.376)	(0.287)
Education	-0.294***	-0.652***	0.024	-0.235*
	(0.087)	(0.137)	(0.126)	(0.110)
Work experience	0.057	0.015	0.087	0.059
	(0.058)	(0.099)	(0.101)	(0.088)
Baseline productivity	-0.003	-0.001	-0.003	-0.004
	(0.003)	(0.004)	(0.003)	(0.003)
Pair fixed effects	YES	YES	YES	YES
Constant	3.849***	6.575***	2.192**	2.867***
	(0.435)	(0.930)	(0.693)	(0.597)

Note: *p<0.05; **p<0.01; ***p<0.001

Supplementary Table 7. *Study 2 Belief in a Just World.*

	Dependent variable:			
	Average	Individual items		
		(1)	(2)	(3)
Participatory meetings	-0.227*	-0.285	-0.421*	-0.004
	(0.112)	(0.217)	(0.241)	(0.237)
Male	-0.275	-0.406	-0.286	-0.127
	(0.205)	(0.318)	(0.328)	(0.225)
Age	0.001	0.0002	0.020	-0.017
	(0.005)	(0.011)	(0.016)	(0.012)
Racial minority	0.087	0.125	-0.126	0.259
	(0.264)	(0.381)	(0.338)	(0.348)
Education	-0.085	-0.160	0.049	-0.148
	(0.067)	(0.108)	(0.118)	(0.113)
Work experience	-0.017	-0.056	-0.061	0.059
	(0.059)	(0.063)	(0.113)	(0.107)
Baseline productivity	0.005*	0.006	0.002	0.006**
	(0.002)	(0.003)	(0.004)	(0.002)
Pair fixed effects	YES	YES	YES	YES
Constant	4.567***	5.513***	3.746***	4.432***
	(0.441)	(0.747)	(0.826)	(0.768)
Note:	*p<0.05; **p<0.01; ***p<0.001			
