# Supplementary material for: Participatory practices at work change attitudes and behavior toward societal authority and justice
Source: Nat Commun. 2020 May 26;11:2633. doi: 10.1038/s41467-020-16383-6 (PMC7250830; doi:10.1038/s41467-020-16383-6)
Supplement: Supplementary file 3 — Reporting Summary [file 41467_2020_16383_MOESM3_ESM.pdf]

nature research

Corresponding author(s): Sherry Jueyu Wu

Last updated by author(s): 2/21/2020

Reporting Summary

Nature Research wishes to improve the reproducibility of the work that we publish. This form provides structure for consistency and transparency in reporting. For further information on Nature Research policies, see Authors & Referees and the Editorial Policy Checklist.

Statistics

For all statistical analyses, confirm that the following items are present in the figure legend, table legend, main text, or Methods section.

n/a	Confirmed	
☐	☒	The exact sample size (*n*) for each experimental group/condition, given as a discrete number and unit of measurement
☐	☒	A statement on whether measurements were taken from distinct samples or whether the same sample was measured repeatedly
☐	☒	The statistical test(s) used AND whether they are one- or two-sided<br>*Only common tests should be described solely by name; describe more complex techniques in the Methods section.*
☐	☒	A description of all covariates tested
☐	☒	A description of any assumptions or corrections, such as tests of normality and adjustment for multiple comparisons
☐	☒	A full description of the statistical parameters including central tendency (e.g. means) or other basic estimates (e.g. regression coefficient) AND variation (e.g. standard deviation) or associated estimates of uncertainty (e.g. confidence intervals)
☐	☒	For null hypothesis testing, the test statistic (e.g. *F*, *t*, *r*) with confidence intervals, effect sizes, degrees of freedom and *P* value noted<br>*Give P values as exact values whenever suitable.*
☒	☐	For Bayesian analysis, information on the choice of priors and Markov chain Monte Carlo settings
☒	☐	For hierarchical and complex designs, identification of the appropriate level for tests and full reporting of outcomes
☐	☒	Estimates of effect sizes (e.g. Cohen's *d*, Pearson's *r*), indicating how they were calculated

Our web collection on statistics for biologists contains articles on many of the points above.

Software and code

Policy information about availability of computer code

Data collection	Data collected from human participants (described below). No software was used for data collection. R Studio is used for all data analysis. Code and data available at OSF.
Data analysis	R Studio (version 3.6.1) is used for all data analysis. All survey items, item groupings, and analyses code can be found at the Open Science Framework (http://bit.ly/2zWf3Ga).

For manuscripts utilizing custom algorithms or software that are central to the research but not yet described in published literature, software must be made available to editors/reviewers. We strongly encourage code deposition in a community repository (e.g. GitHub). See the Nature Research guidelines for submitting code & software for further information.

Data

Policy information about availability of data

All manuscripts must include a data availability statement. This statement should provide the following information, where applicable:

- Accession codes, unique identifiers, or web links for publicly available datasets
- A list of figures that have associated raw data
- A description of any restrictions on data availability

We report how we determined our sample size, all data exclusions (if any), all manipulations, and all measures in the study. All survey items, item groupings, and analyses can be found at the Open Science Framework (http://bit.ly/2zWf3Ga). Study materials and additional analyses can be found in the supplementary material.

Field-specific reporting

Please select the one below that is the best fit for your research. If you are not sure, read the appropriate sections before making your selection.

☐ Life sciences ☒ Behavioural & social sciences ☐ Ecological, evolutionary & environmental sciences

For a reference copy of the document with all sections, see nature.com/documents/nr-reporting-summary-flat.pdf

Behavioural & social sciences study design

All studies must disclose on these points even when the disclosure is negative.

Study description

Two quantitative field experiments (China and United States)

Research sample

Study 1: Chinese factory workers in the city of Suzhou, China. We sampled all the sewing groups in the factory (65 groups, Nworkers = 1,752; 93.6% female; mean age = 32.5 years, ranging from 18 to 53). The sample is representative of Chinese factory workers in the manufacturing industry, but may not be representative of the Chinese national population.
Study 2: American university staff members, United States. We sampled the departmental groups consented to participate in the study (32 groups, 172 individual staff members, 78% female, 22% male; 80% identified as White or European-American; mean age = 50 years, ranged from 25 to 88 years). The sample is representative of this University's staff groups, but may not be representative of the American national population.

Sampling strategy

In Study 1, we sampled all 65 sewing groups in the China branch of a multinational apparel manufacturer. We randomly assigned the 65 work groups (Nworkers = 1,752; 93.6% female; mean age = 32.5 years, ranging from 18 to 53) to participate in a weekly morning participatory meeting (referred to as participatory meetings condition or treatment condition), or to have an observer attend the usual morning meeting (referred to as observer condition or control condition) once per week for six weeks. To randomize, we used a non-bipartite matching scheme (Beck, Lu, & Greevy, 2015) (see Supplemental Information for matching procedure and code). Post-hoc power analysis indicates that the achieved power given our sample size and average effect size was 0.99.

In Study 2, we recruited 32 academic staff groups and randomly assigned the staff groups to participate in a weekly morning participatory meeting (participatory meetings or treatment condition) or continue with their status-quo meetings (control condition). Thirty-two academic departments' administrative staff groups or 172 individual staff members participated in the study (78% female, 22% male; 80% identified as White or European-American; mean age = 50 years, ranged from 25 to 88 years). Post-hoc power analysis indicates that the achieved power given our sample size and average effect size was 0.82.

Data collection

Survey data were collected four weeks (or two weeks as in Study 2) after the end of the intervention. Pen and paper were the only instruments used in the data collection procedure. Research assistants, who were blind to experimental condition and study hypotheses, were present during the data collection.

Timing

Study 1: 4/4/2016 - 8/9/2016
Study 2: 9/1/2017 - 1/26/2018

Data exclusions

No data were excluded.

Non-participation

No participants dropped out/declined participation in study 1. No participants dropped out in study 2; 32 out of 68 staff groups consented to participate in the experiment.

Randomization

The unit of randomization is on the group level. All groups are intact existing groups. We randomly assigned work groups into treatment condition and control condition (see above).

Reporting for specific materials, systems and methods

We require information from authors about some types of materials, experimental systems and methods used in many studies. Here, indicate whether each material, system or method listed is relevant to your study. If you are not sure if a list item applies to your research, read the appropriate section before selecting a response.

Materials & experimental systems

n/a	Involved in the study	
☒	☐	Antibodies
☒	☐	Eukaryotic cell lines
☒	☐	Palaeontology
☒	☐	Animals and other organisms
☐	☒	Human research participants
☒	☐	Clinical data

Methods

n/a	Involved in the study	
☒	☐	ChIP-seq
☒	☐	Flow cytometry
☒	☐	MRI-based neuroimaging

Human research participants

Policy information about studies involving human research participants

Population characteristics	See above.
Recruitment	In Study 1, we recruited all 65 sewing groups (1752 workers) in the China branch of a multinational apparel manufacturer. In Study 2, we recruited 32 academic administrative staff groups (172 staff members; out of 68 administrative groups) in a private university in the United States. We were not particularly concerned with potential selection bias: participating and nonparticipating groups did not differ in relevant characteristics such as group size, academic division, or group composition. We also asked 7 independent raters from the university's central offices who have regular and frequent contact with ALL staff groups to rate the average staff performance and found no difference between those who chose to participate in the experiment and those who did not.
Ethics oversight	Princeton University Institutional Review Board.

Note that full information on the approval of the study protocol must also be provided in the manuscript.
